# The Impact of Perceived Stigma, Quality of Life, and Spiritual Beliefs on Suicidal Ideations among HIV-Positive Patients

**DOI:** 10.1155/2018/6120127

**Published:** 2018-03-26

**Authors:** Nooshin Zarei, Hassan Joulaei

**Affiliations:** ^1^Shiraz HIV/AIDS Research Center, Institute of Health, Shiraz University of Medical Sciences, Shiraz, Iran; ^2^Health Policy Research Center, Institute of Health, Shiraz University of Medical Sciences, Shiraz, Iran

## Abstract

**Background:**

Suicidal ideation is considered a major health problem associated with HIV/AIDS. Suicide rates among people living with HIV/AIDS (PLHA) are more than three times higher in the general population and that is a significant difference. This study aimed at investigating the related factors of suicidal ideations among HIV-positive patients in Southwest Iran.

**Study Design:**

A cross-sectional study.

**Methods:**

351 adult volunteer HIV-infected patients that referred to the Voluntary Counseling and Testing (VCT) center in the south of Iran were evaluated based on convenience sampling. Data was collected utilizing a structured questionnaire from March to August 2015.

**Results:**

Over the six months prior to the study, 15.4% of the entire sample had been diagnosed with suicidal ideation. There was a significant correlation between the quality of life, spiritual beliefs, perceived stigma, and age with suicidal ideation. Suicidal ideation is significantly different in terms of gender and marital status. Perceived stigma and spiritual beliefs showed the highest effect on suicidal ideations, respectively.

**Conclusions:**

Having religious beliefs due to accelerating psychological adaptation can motivate HIV patients to survive and have also been considered effective in preventing women from suicide. Perceived stigma and quality of life are other factors that should be taken into consideration as key elements in suicide preventive programs.

## 1. Introduction

Suicidal ideation is considered a major health problem associated with HIV/AIDS [1, 2]. It is more common than suicide attempt and increases the risk of suicidal attempt and completed suicide [3]. Suicide rates among people living with HIV/AIDS (PLHA) are more than three times higher in the general population [4]. According to a systematic review study, the prevalence of suicidal ideation among patients with HIV varied from 13.6 to 31.0% until January 2015 [2]. The prevalence of suicidal ideation in the general population of Iran is 10 to 12.7% [5, 6].

Previous studies showed that younger and low educated PLHA have more suicidal ideations [7, 8]. Those with suicidal ideation reported high levels of depressive symptoms and mental problems [9–11], and this is positively linked with higher levels of internalized AIDS stigma among PLHA [12].

Consequently, hopelessness and negative evaluations lead to low engagement in treatment and health services and a tendency to ignore coping strategies to deal with such symptoms which in turn lead to an increase in social isolation and more risk of suicide [13]. The negative correlation between suicidal ideation and spiritual beliefs implied the importance of spirituality in coping with different health problems and HIV as well [14, 15]. Patients with a poorer quality of life (QoL) were more likely to experience depression and suicide risk than patients with higher QoL that is associated with daily functioning [16–18]. In addition, the consideration of suicidal ideations in HIV/AIDS is important not only because it predicts future attempted suicide, but also because it has been associated with poor quality of life and poor adherence with antiretroviral therapy [19, 20].

Although knowledge about the prevalence of suicidal attempts is important, suicidal ideation is more relevant and significant to understand. Perhaps by understanding the tendencies of people before committing suicide, some measures can be taken to prevent suicide. Few studies have been carried out on suicidal attempts and ideations among PLHA in Iran that consider effective factors such as spirituality, but perceived stigma as a key determinant factor is neglected [21, 22].

Due to the culture of the Iranians, stigmatized attitude is associated with more prominent role of guilt and knowing the patients involved in immoral behavior. Therefore, internal stigma and discrimination are higher in Iran in comparison with other countries which can lead to more suicidal ideation in HIV-positive individuals [23].

Therefore, this study was designed to investigate the impact of perceived stigma, quality of life, spiritual beliefs, and some other related factors on suicidal ideations among HIV-positive patients.

## 2. Methods

The population of this cross-sectional study consisted of all patients living with HIV/AIDS in Fars province. Although the exact number is unknown, according to the latest reports of the Deputy of Health in Shiraz University of Medical Sciences in September 2013, the total number of PLHA in Fars province of Iran was 4410; of them, 81% were male and 19% female.

People in the community are urged to undergo HIV testing in three ways; community awareness and education, venues such as Drop-In Center (DIC) to encourage sex workers, injecting drug users and other high-risk groups for voluntary testing, and the peers. In spite of implementing different programs to drive people for voluntary HIV testing, most of them were anonymous and not readily available. That is why respondents were selected from patients who were referred to the Voluntary Counseling and Testing (VCT) Center.

So, 351 adult volunteer HIV-infected patients referring to the VCT center in Fars province, Iran, were enrolled in the study. The questionnaire was used as a data collection tool and because some subjects were less educated, an expert who worked at the VCT filled out the questionnaires to communicate better with the patients in order to ensure accurate input of answers. The data was collected from March to August 2015 based on convenience sampling. Ethical approval was obtained from the Ethics Committee of the Shiraz University of Medical Sciences.

Inclusion criteria were HIV-positive patients in Fars province who referred to VCT center and mandatorily completed an informed consent form. Patients who did not possess any of these criteria were excluded from the study.

### 2.1. Research Tool

A questionnaire was designed and used as a tool for measuring and collecting the data. The questionnaire included demographic information, 7 items for perceived stigma, 21 items for measuring the quality of life, 13 items for spiritual and religious beliefs, and 4 items for measuring suicidal ideations using Likert's scale. For this scale, we asked participants if they had these ideations within six months prior to the study. Moreover, the interaction of the personnel with patients was also evaluated.

QoL was measured with the ACTG SF-21 which was originally adapted from the Medical Outcomes Study HIV Health Survey (MOS-HIV), a measure with well-established reliability and validity [24]. The ACTG SF-21 consisted of 8 domains: general health perceptions, physical functioning, role functioning, social functioning, pain, mental health, cognitive functioning, and energy/fatigue. Across all domains, higher scores indicate better quality of life. To evaluate stigma and discrimination, 12 items (four-choice question, 7 for internal and 5 for external/social stigma) were asked that was used by USAIDS in 2006 [25]. The range of total score was 12 to 48. Scores of 12–22.6, 22.6–33.26, and 33.26–44 were labeled as low, moderate, and high, respectively.

Despite a valid Persian questionnaire for measuring religious beliefs [26], we still have no valid questionnaire for measuring spiritual beliefs. To ensure the validity of the selected items, we solicited experts' views. A pilot study was carried out in order to gain the internal validity of the questionnaire and to determine the participant's full understanding of the questions or not. The range of total score of religious beliefs of the participants in 13 items using 5-point Likert scale. Each item score was varied between 0 (never) and 4 (always).

Although the scales were used in national projects, and the valid ACTG SF-21 questionnaire was translated in Persian, the items were pretested on 60 subjects of the study population after customization. The Cronbach's alpha was used to ensure the reliability of the items. The amounts of internal/perceived stigma, spiritual beliefs, and suicidal ideations were obtained, 0.743, 0.771, and 0.821, respectively, which were acceptable according to the sample volume of the pretest. In order to check on face validity, the surveyed items are sent to experts to obtain suggestions for modification. There was an attempt to select items that are based on common sense and seem right to the readers.

### 2.2. Data Analysis

The data were analyzed using SPSS software, version 21. Descriptive statistics such as mean, standard deviation, and frequency distribution tables were applied to describe the results. To compare suicidal tendencies among different genders, we used independent sample *T*-test. A binary logistic regression analysis was applied to identify the predictive variables of suicidal ideation. Bivariate relationships were examined between suicidal ideation, perceived stigma, quality of life, spiritual beliefs, age, and educational level through calculation of spearman correlation coefficients. Finally, a multiple regression model was run to assess the importance of each variable in explaining the suicidal ideations.

## 3. Results

Majority of the participants in the current study were married (40.5%) with education level (67.3%) less than high school diploma. Majority of them (56.1%) were at the low level of self-assessed socioeconomic status. In terms of the employment status, although all of the participants were in the working age, most of them were unemployed (70.1%) (Table 1).

As to the routes of HIV transmission, 186 (52.9%) participants declared shared injection drug use, 92 (26.2%) had sexual contact with their infected spouse, 47 (13.4%) experienced extra-marital and unsafe contacts, and 26 (7.4%) pointed to the other routes such as tattoos.

Overall, 54 (15.4%) of the entire sample had suicidal tendencies over six months prior to the study. Males reported more suicidal tendencies (*M* = 10.31, SD = 23.99) than females (*M* = 4.32, 16.51), and the difference was statistically significant (*t* = −2.712, *p* = 0.007).

It is also important to note that spiritual beliefs among females were more (*M* = 10.53, SD = 1.24) than males (*M* = 9.37, SD = 2.01) and the difference was significant (*t* = 6.63, *p* = 0.0001). As to the quality of life scale, the data analysis showed that there was a significant difference between men and women (*t* = 5.25, *p* = 0.0001). The score of this scale was higher for females (*M* = 291.97, SD = 145.13) in comparison with males (*M* = 204.29, SD = 143.32). There was also a significant difference in duration of HIV infection between patients with suicidal ideations (5.10 ± 2.59) and without the ideations (4.25 ± 2.57) (*t* = 2.18, *p* = 0.044).

The suicidal ideation was significantly different in terms of marital status, as well (*F* = 4.42, *p* = 0.005). The score was higher for single patients (*M* = 1.48, SD = 1.009), in comparison with married ones (*M* = 1.21, SD = 0.769). In addition, the suicidal ideation was different according to different socioeconomic status (*f* = 8.003, *p* = 0001).

According to Spearman correlations, significant negative correlations between the QoL and spiritual beliefs with suicidal ideation were recorded, while the correlation between perceived stigma and suicidal ideations was positive. All the mentioned correlations were significant even after controlling for duration of HIV infection as a possible confounder (Table 2).

A significant negative correlation between all the QoL dimensions and suicidal ideation was recorded based on the correlation tests (Table 3).

The results of linear multiple regression model indicated the effect of each variable controlling for other explanatory variables. Perceived stigma and spiritual beliefs showed the highest effect on suicidal ideations, respectively (*β* = 0.598, −0.155). Determination coefficient for the model (*R*^2^ = 0.42) shows that 42 percent of variance in suicidal ideations was explained by the variables examined (Table 4).

## 4. Discussion

Considering the fact that suicidal ideation is a health problem associated with HIV, this study was conducted to investigate some of the related factors of such ideations. According to the results of the present study, 15.4% of PLHA had suicide ideations over the six months prior to the study, whereas in the general population this rate is between 10 and 12.7% [5, 6]. It is expected that the prevalence of lifetime suicidal ideation is more dramatic, while a person who lives with HIV virus is under intense internal pressure. This fact indicates the importance of considering the influencing factors that would reduce the incidence of suicide among PLHA.

The results showed that younger PLHA have more suicidal ideation that is consistent with previous studies [8]. In spite of the results of previous studies that showed a significant relationship between the lower level of education and suicide ideation [27], we did not find any relationship between these factors. One of the possible reasons for such difference is that the studied sample was relatively homogenous in terms of education and most of them had low literacy.

The results showed that patients with suicidal ideations had more length of time since HIV diagnosis. Previous studies found different results; although a study in Iran found no significant relationship between suicidal ideation scores and time from HIV infection diagnosis, a study in China found that most HIV-positive individuals made their first suicidal attempt/ideation immediately after their HIV diagnosis within a month [21, 28]. The possible reason for such a difference is that we had no HIV-infected patient who is in the early stages of coping with an HIV diagnosis in the current study. Therefore, we compared the patients who have been infected for at least two and a half years. The reasonable reason is living longer with HIV may increase posttraumatic stress disorder and depression symptoms [29, 30].

A significant point of consideration in this study is that males reported more suicidal ideations than females, which is not inconsistent with the previous results [27]; and there are probable reasons. Among the studied samples, most of them were married. The results revealed a significant difference in suicidal ideations between single and married patients. Factually, having a family and being in a family union increase their motivation to survive, especially because the majority of them had children and felt responsible towards them. The other reasons can be the higher quality of life, on the one hand, and more spiritual beliefs among the studied women, on the other hand. According to the previous studies, religion helps the patients to cope better with the suicidal crisis [31]. It also seems that having religious beliefs due to increased life expectancy and motivation of survival can be effective and prevents patients from committing suicide.

The significant negative correlation between suicidal ideation and spiritual beliefs implied the importance of spirituality and such beliefs which are consistent with other studies and can accelerate with psychological adaptation among PLHA [14, 32].

Suicidal ideation is negatively correlated with QoL both overall and each of the QoL subscales. Previous findings also confirm that patients with a poorer quality of life were more likely to experience suicide risk than patients who had higher QoL [16, 33].

Perceived stigma was identified as an important factor for suicidal ideations in this study as well. The results of a study that was done by Amiya et al. showed that depressive symptoms, rather than suicidal ideations, were positively linked with higher levels of internalized AIDS stigma among PLHA [12]. According to the results, both perceived stigma and spiritual beliefs were the most effective factors of suicidal ideation which should be considered more in the preventive strategies to reduce suicidal ideations.

Considering the high prevalence of perceived stigma in HIV-infected patients in the same context [34], harm reduction programs and counseling services with more emphasis on reducing internal HIV-stigma and increasing spiritual beliefs should be implemented to decrease the possible suicidal ideations and attempts among HIV patients.

## Figures and Tables

**Table 1:** Demographic characteristics of the participants.

Sociodemographic characteristics	*N* (%)	*p* value
*Age* ^*∗*^ (years)	39.53 ± 7.5	0.120
*HIV infection duration* (years)	5 ± 2.61	0.044
*Gender*		
Male	241 (68.7)	0.007
Female	110 (31.3)	
*Marital status*		
Single	109 (31.1)	0.005
Married	142 (40.5)	
Divorced	74 (21.1)	
Widowed	26 (7.4)	
*Education level*		
Illiterate	7 (2)	0.083
Less than diploma	267 (76.1)	
Diploma	60 (17.1)	
University degree	17 (4.8)	
*Occupational status*		
Employed	102 (29.0)	0.003
Unemployed	246 (70.1)	
Retired	3 (0.9)	
*Socioeconomic status*		
Lower class	193 (56.1)	0.001
Lower middle class	127 (36.9)	
Upper-middle class	24 (7.0)	
Upper class	0 (0.0)	

^*∗*^Mean ± SD.

**Table 2:** The correlations between suicidal ideation and QoL, perceived stigma, and spiritual beliefs.

	Suicidal ideation	QoL score	Perceived stigma	Spiritual beliefs
Suicidal ideation	1.0			
QoL score	−0.258^*∗∗*^	1.0		
Perceived stigma	0.543^*∗∗*^	−0.496^*∗∗*^	1.0	
Spiritual beliefs	−0.265^*∗∗*^	0.206^*∗∗*^	−0.133^*∗*^	1.0

^*∗∗*^Correlation is significant at the 0.01 level (2-tailed). ^*∗*^Correlation is significant at the 0.05 level (2-tailed).

**Table 3:** The correlations between suicidal ideation and QoL dimensions.

	Suicidal ideations	General health	Physical functioning	Role functioning	Social functioning	Cognitive functioning	Pain	Mental health	Energy/fatigue
Suicidal ideations	1.0								
General health perceptions	−0.303^*∗∗*^	1.0							
Physical functioning	−0.118^*∗*^	0.467^*∗∗*^	1.0						
Role functioning	−0.184^*∗∗*^	0.542^*∗∗*^	0.470^*∗∗*^	1.0					
Social functioning	−0.167^*∗∗*^	0.563^*∗∗*^	0.436^*∗∗*^	0.600^*∗∗*^	1.0^*∗∗*^				
Cognitive functioning	−0.228^*∗∗*^	0.514^*∗∗*^	0.514^*∗∗*^	0.468^*∗∗*^	0.530^*∗∗*^	1.0			
Pain	−.140^*∗∗*^	0.550^*∗∗*^	0.512^*∗∗*^	0.520^*∗∗*^	0.512^*∗∗*^	0.494^*∗∗*^	1.0		
Mental health	−0.278^*∗∗*^	0.614^*∗∗*^	0.439^*∗∗*^	0.459^*∗∗*^	0.497^*∗∗*^	0.509^*∗∗*^	0.478^*∗∗*^	1.0	
Energy/fatigue	−0.196^*∗∗*^	0.553^*∗∗*^	0.483^*∗∗*^	0.455^*∗∗*^	0.481^*∗∗*^	0.456^*∗∗*^	0.445^*∗∗*^	0.580^*∗∗*^	**1.0**

^*∗∗*^Correlation is significant at the 0.01 level (2-tailed). ^*∗*^Correlation is significant at the 0.05 level (2-tailed).

**Table 4:** The linear multiple regression for suicidal ideations as a dependent variable.

Variables	Std. error	Beta	*t*	*p* value
Age	.002	−.087	−1.841	0.067
Social class	.027	−.115	−2.412	0.016
Male	.041	.011	.199	0.842
Employed	.040	−.002	−.034	0.973
Single	.041	.011	.194	0.846
Divorced/widowed	.038	−.016	−.322	0.747
HIV infection duration	.049	.024	.515	0.607
Perceived stigma	.003	.596	11.662	0.001
Quality of life	.000	.092	1.604	0.110
Religious beliefs	.009	−.140	−2.949	0.003

## Additional Points

*Limitations*. Respondents were selected from the patients who had referred to the Voluntary Counseling and Testing (VCT) Center and benefited from the consulting services of the center. Therefore, lack of access to the patients outside VCT was a limitation of the current study.

*Suggestions for Future Studies*. The interventional studies through counseling services for HIV-infected patients should be considered in future studies to decrease perceived stigma as a key factor of suicidal ideation among the patients.
